# Why scientists, academic institutions, and investors fail in bringing more products to the bedside: the Active Compass model for overcoming the innovation paradox

**DOI:** 10.1186/s12967-021-02726-4

**Published:** 2021-02-04

**Authors:** Yaron Ilan

**Affiliations:** Department of Medicine, Hebrew University-Hadassah Medical Center, POB 1200, IL91120 Ein-KeremJerusalem, Israel

**Keywords:** Innovation, Physician scientist, Translational research, Startups

## Abstract

The vast majority of good science and excellent ideas do not translate into products. Many good products that have the potential to assist in diagnosis and therapy do not mature into everyday care. This often becomes a source of frustration for innovators, academic institutions, companies both small and large, and investors. The “innovation paradox” , wherein excellent ideas and good science fail to reach the bedside, is a major challenge. This study presents the Active Compass model as a way to overcome this obstacle. The model is designed to assist projects at early stages by redirecting and reshaping them in a way that increases their chances of reaching the markets. The model is based on the use of next-generation translational research and on creating differentiators at the early stages of development. The proposed model’s implementation by innovators, scientists, technology transfer offices, academic institutions, analysts, and investors can help move forward high-potential projects to improve the quality of life and alleviate the burdens of disease.

## The problem: The "innovation paradox," wherein multiple good ideas and data do not mature into products

**“**We sit on golden eggs; how come they are not being turned into products (and profits)?” This question is commonly asked by scientists, entrepreneurs, directors of academic institutions, investors, and venture capitalists. Excellent ideas, great laboratory data, and startling clinical observations commonly fail to materialize into products in a way that can improve the quality of life and alleviate diseases. The number of new ideas, new molecules, pre-clinical data, and clinical findings that reach the licensing stage or mature into a product is extremely low [1]. As many as 10,000 compounds may be considered and whittled down to just 10, which could theoretically interfere with the disease process. For every 25,000 compounds that start in the laboratory, 25 are tested in humans, five make it to market, and just one recoups what was invested [2]. The current estimate is that less than 10% for those that reach the startup stage are turned into a product [3, 4]. Of the companies that survive, most do not bring a sustainable product to market. This “innovation paradox,” wherein the vast majority of good science and excellent ideas do not translate into products, is frustrating for innovators, institutions, and investors.

## Solution: The Active Compass model, implemented in the first steps of development

Overcoming the innovation paradox involves multiple factors at different stages of product development that bring a final product to the end-user [5–7]. This study focuses on the initial stages of taking an idea or early-stage result to the bench, and from the bench to a proof of concept. The Active Compass model is designed to redirect and reshape the innovation at an early stage and is based on two cornerstones: the next-generation translational research concept and the objective of creating a differentiator. Together, these two cornerstones serve as the basis for a schematic model that can assist in overcoming several of the major barriers confronting those who walk the winding and obstacle-strewn road of success.

Importantly, although this model is useful for the early stages of development, it also has many implications for later stages. In fact, several of the barriers confronted at later stages, some of which cause ventures to fail, can be prevented by redirecting the innovation at an earlier stage using the proposed model.

## Next-generation translational research: Translating the innovation into the right platform by looking at end-users' first

The first cornerstone of overcoming the innovation paradox is looking differently at translational research. Translational research involves efforts to build on basic scientific research, new therapies, medical procedures, or diagnostics. Translational research involves the multidisciplinary integration of basic research, patient-oriented research, disease-oriented research, and population-based research, aimed at improving diagnosis and prognosis [8]. Much translational research is based on physician-scientists’ bringing of ideas from bench to bedside [9, 10]. Physician-scientists invest significant time in scientific research, mainly inpatient or disease-oriented research, and spend less time in direct clinical practice [11, 12]. The current physician-scientist model requires them to test new concepts or in vitro data in disease models or asks them to design a prototype. This has proven to be insufficient for a large proportion of projects [13–16].

Next-generation translational research is based on the notion that translating ideas, preliminary observations, and initial results in a broad and applied sense requires a better understanding of the problem to be solved and the practicality of the solutions offered. Designing next-generation translational research requires that the targets and the questions the research and product are expected to answer be redefined. Several methods for implementing next-generation translational research are outlined below.

Next-generation translational research must begin at the end, by determining not only who is going to pay for the product but also whether end-users are going to use it. Once it is clear that someone will use the product and somebody else will pay for it, the next step is to redefine the need. Next-generation translational research focuses on the end-user in a broader sense. The focus is on the clinical outcome; the likelihood of adoption by patients, their caregivers, and medical institutions; and payer support.

While a highly significant effect (e.g., curing over 90% with no side effects) can always provide an answer to all issues, this is highly unlikely to occur for most drugs. Finding an answer may require sub classifying a disease into categories or sub classifying the patient population into segments. Similarly, if the problem to be solved concerns the partial success of an existing solution, next-generation translational research will investigate combination therapy, which is commonly used with checkpoint inhibitor combination therapy [17, 18]. Similarly, if side effects from checkpoint inhibitors present an obstacle to their use, alleviating the side effects rather than focusing solely on the clinical outcome of the combined technology may offer an answer to an unmet need, which is highly likely to be used by patients and supported by clinicians and payers alike.

Next-generation translational research involves predefining market needs, identifying unmet niche needs, staying away from overcrowded areas where there are already multiple adequate solutions, or identifying prominent differentiators between highly jammed areas. Real unmet needs are not always easy to identify. Detecting them requires looking at problems from different angles. What seems to be a need for clinicians may not necessarily be a real market need and vice versa. Markets and companies may develop answers to needs they believe exist but which clinicians or medical institutions are not prioritizing. Similarly, needs that are accepted by clinicians and companies may not necessarily be adopted by patients. For example, “minor” side effects that are not considered serious by clinicians may become prohibitive to patients. Time-consuming procedures such as preparing a syringe for medication self-administration are associated with low adherence. End-user compliance is a part of next-generation translation.

An additional factor that is highly relevant for next-generation translational research is the often-ignored factor of the body’s compensatory response to triggers induced by a drug or any type of intervention [18, 19]. Ignoring this problem is associated with lower effectiveness in many drugs used for chronic conditions, sometimes in up to 40% of patients, such as those with epilepsy or depression, where many patients develop drug resistance [20, 21]. Thus, next-generation translation research is required to provide a way to deal with compensatory responses [22–30].

Next-generation translational research needs to consider intra- and inter-patient variability in responses. This problem can lead to the failure to respond to therapy among segments of patients who are not always easily identified [29]. This requires redesigning the product and the research.

Using next-generation translational research may require going back to the bench for new experiments or using a different data-analysis method to obtain answers to new questions.

## Creating a differentiator: understanding where your competitors are and will be

The second cornerstone of overcoming the innovation paradox is identifying potential differentiators at very early stages of the development process. The drug and device development environment is highly competitive. Needs are usually well-established, and niches are no longer hidden. Thus, differentiators must be identified as early as possible to redirect projects toward paths that can lead to the end-user and the market. What are the advantages and disadvantages of the proposed product compared with existing solutions and those that are being developed and are expected to reach the market in the coming years?

Creating a differentiator is complicated. It requires an in-depth understanding of the implications of the scientific results as well as of the market. Several common methods of creating differentiators are outlined below.

Knowing your competition is the first step in finding differentiators. Commonly used differentiators include the data themselves. They are used to find a new mechanism, identify a new target or niche that no one has targeted, develop superior efficacy or likelihood of end-user adoption, deploy the right branding (“comes out of Harvard”), and emerge first in the race. However, as exemplified below, none of these may be sufficient.

Differentiating the product is easy if the project involves the first and only drug for a certain disease. Otherwise, as in most cases, the product needs to be at least non-inferior to, and preferably better than, existing solutions. One important element may be time. If someone is already ahead in development by several years and the proposed product is inferior, it is highly unlikely that you will get it to market. However, if the product solves a problem that is not answered by current competitors, the fact that a competitor is paving the way toward a niche may be useful. An improved product may be one with better efficacy or fewer side effects, or that targets segments of patients to which the competitor is less likely to provide an answer, or that can be used in combination with the competitor. Each kind can help one deal with the “they are ahead of me” problem.

It is important to identify which new mechanisms are being explored by others as ways for products in development to target diseases. An extensive search of the ClinicalTrials.gov website can assist in finding ongoing trials. It is important to conduct an in-depth patent search for technologies being developed for the same or similar indications. Identifying competitors requires changing strategies, and even changing targets at very early stages of development.

There are multiple ways to create a differentiator. It is commonly claimed that the technology itself is the differentiator. Most innovators fall in love with their data and believe it to be a differentiator. However, the data are often insufficient. If the new technology, though somewhat better and even cheaper, faces a giant pharma company playing in the same field, it is highly unlikely to succeed. It may take much time and expense that no one will be willing to invest to penetrate the wall created by an existing, though lower-level, product.

Intellectual property (IP) is considered by many to be a differentiator. In reality, IP is a simple translation of the data into legal terms. However, neither the data nor the IP based on them are always good enough differentiators. In fact, most patents end up without a product in the market. Patents are limited by the boundaries created by the data themselves. They cannot protect something that does not exist or is only vaguely implied by the results. Patent attorneys, try as they might, cannot bridge the gap between the data presented to them and the market.

Identifying a niche and directing the results toward it can serve as an excellent differentiator. However, this is not always an easy task. In many cases, the niche that is believed to have been “discovered” is already taken. Finding a “niche within a niche” may not be sufficient, as it may end up being too small a market.

Branding can be a differentiator. This makes life easier for top institutions, famous researchers, and those who can create a differentiator based on a publication in a high-ranking journal. Data generated by a high-level institution will outperform similar data generated by a mid-level laboratory.

Having more than one major differentiator is significant. Having multiple minor differentiators is also an option, but this is less effective, as each minor advantage is usually incapable of standing alone against the competitors. If no differentiators exist, the project needs to be redirected in such a way that it can have clear ones. Looking into the competitors may highlight new directions that can be taken for the project. Unfortunately, if these cannot be identified, closing down the project is always better than dragging it further based on faint hopes and investing wasted effort and energy.

## Technology transfer offices, investors, and venture capitalists fail to make the most out of what they have: focusing on what is important and not on technicalities

Technology transfer offices, analysts, and venture capitalists fail to turn most of their projects into valuable products. This often happens because they look at only one side of the coin: either the market need or the data. They fail to bridge the gap. Holding the data up as the torch in front of the project is similar to using IP as the torch. For most projects, turning the data, as good as they are, into a product is insufficient to ensure adoption by end-users, clinicians, and patients. In many cases, the data are not translated into the product (in the broad sense of the word) and/or do not provide a sufficient differentiator. Those involved in making the decisions may focus on technical issues. They can take the side of the inventor and focus on the data, spending most of their time on IP, licensing agreements, and contracts, which usually never mature into products. Lawyers are the sole beneficiaries of these processes. The number of licensing agreements is far from equal to the number of products. Milestones, one of the most common parameters used by institutions, rarely lead to product sales.

Investors and analysts often consider what they believe is the market need and the potential return on investment. They analyze PowerPoint and Excel documents, which are often based on unrealistic hypotheses. The fact that 9% of the population suffers from diabetes does not mean that the data can be turned into another anti-diabetes unicorn. Investors and analysts tend to consider how consumers will end up looking at the product. They fail to see the patient or consider whether clinicians and their institutions will end up using it. They spend much of their time criticizing presentations and what they believe to be redirecting, changing numbers on Excel sheets, and inquiring into production costs, sale prices, and profits. Their view of the competition is narrow in the sense that they look at other runners and not at the customers. They often ignore end-users and their needs.

The Active Compass model refocuses the attention of all those involved in the early stages of product development onto the target-the end-users-not only on their needs but also on the question of whether they are actually going to use the product. Reshaping the product means looking at competitors from every angle. The same insulin but administered in an easier way or more cheaply can serve patients much better than can a drug that works via a novel mechanism. A mode of administration that is easier for patients is an excellent differentiator in this case. An oral drug that can replace injections can have an immediate benefit if its side effects are tolerable.

Focusing on next-generation translations and differentiators should be the priority. It is easy to focus on technicalities such as by spending much time on presentations, Excel sheets, contracts, and IP. They keep the considerations superficial and prevent a focus on what is important for the project to succeed.

## Implementing the Active Compass model: all the people involved

Implementing the Active Compass model solution starts and ends with the people involved. The key is selecting the right people to guide brilliant thinkers, scientists, entrepreneurs, and investors who wish to take the next step from their bench toward the market, or who seem to like the project and want to invest. The Active Compass model aims to select and generate a new profession: Active Compass Guides. These guides are asked to implement the next-generation translational research and the differentiator models for the project at the early stages of development.

Medical doctors (MDs) who spend most of their time conducting inpatient care do not understand the relevant markets and competitors, and most MDs who leave the bedside behind do not necessarily understand real clinical needs. Putting both of them at the same table sometimes leads to a dialogue among the deaf: Neither side has a real understanding of the other. Once they reach an agreement and start marching on the long way to the market, however, it is very difficult to make a U-turn.

Drug and medical device development is a long process that can take many years. Very few people working in the healthcare system and industry are experienced in taking a product from A to Z. Even the most experienced of them have taken only a few steps in that process and can look only one or two steps ahead. When standing at the starting line, they see the finish line only vaguely. Adding people to the team at different stages of development does not resolve the problem. The further a project advances in a certain direction, the harder it is to change it. Those who join the project later merely help push it in the same direction. Therefore, using a mixed team comprised of clinicians, investors, regulatory experts, and other professionals-the most common team type-is insufficient for overcoming the problem.

Identifying active compass guides is difficult, as these people are rare. The preferred guides are clinically active MDs with expertise in their field, parallel experience, and visions of real needs and markets. They must show an in-depth understanding of their competitors and must be able to see the future of their target and solution. They need to have a broad enough view of the field’s short- and long-term directions and can also assist in fund raising for the selected projects. They must have a comprehensive knowledge of the different mechanisms of action, the different drugs and devices, and the failures and successes over the last few years in the field. Past experience is very important.

Guides are difficult to find, and they must start being generated. If one cannot be identified, a guide can be created by combining two people. One way of overcoming this problem is to create small teams of one to three "less-experienced” guides. Selecting guides who have failed multiple times is often better than selecting a “star” who made it once.

The Active Compass Guide must be part of the developing group, which will also include investors. Guides should not play the role of “outside consultant.” An ideal way to generate a commitment is to provide the Guide equity in the project. Rather than charging by the hour to give advice, the Guide then becomes one of the keystones of the project. Greed among entrepreneurs and investors leads to a lack of understanding of what is required at the early stages of the project.

Once the right people are in place, the project must be reshaped. This may require only the restructuring of the existing data or a new emphasis on points that had been ignored. Alternatively, it may require going back to the bench or using different models or different targets. In some cases, the Active Compass will direct the project to a cemetery; if there is no niche or differentiator, it is better to stop early.

**Fig. 1:**
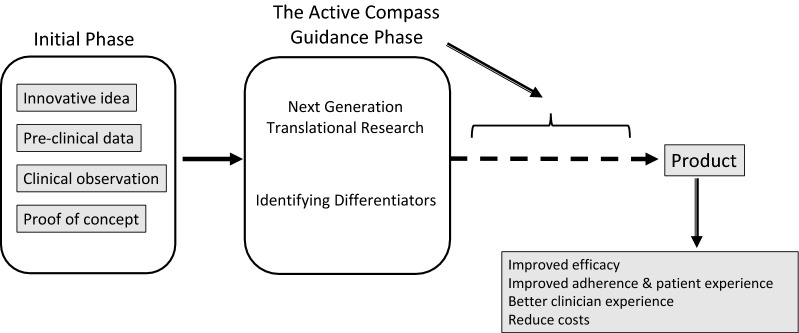
A schematic presentation of the Active Compass model for dealing with the innovation paradox. In the initial phase, clinical observations, pre-clinical data, and innovative ideas are presented. The Active Compass Guidance phase involves the two cornerstone processes of next-generation translational research and differentiator identification. This phase is expected to pave the way to the final product

Figure 1 schematizes the reshaping of an idea, pre-clinical data, or clinical observation using the Active Compass model. If the project is restructured from the early stages based on a next-generation translation and with profound differentiators, it will have already overcome many of the obstacles involved in the next steps.

The Active Compass model comprises the two cornerstone processes of next-generation translational research and differentiator creation. This model requires much time and energy; it may take months, and even years. It involves a brainstorming session and in-depth analysis of multiple parameters, as outlined above. It is a crucial investment. Taking shortcuts in this process may lead to disaster. Most importantly, the Active Compass model is not a magic bullet solution. It is an ongoing process that should accompany any project throughout all its stages of development. Most products involve years of development, during which targets and needs change, competitors fail or succeed in areas that were not anticipated, and new advantages and disadvantages of the product are revealed. Careful, continuous monitoring is needed. The project must adapt to multiple changes. This may require taking several steps back, redirecting, identifying new targets, or even deciding on early termination.

## Summary

The innovation paradox is a source of deep frustrations among all those associated with drugs and medical device development. The most important consequence of this paradox is that many excellent ideas and data do not turn into products that can benefit patients, improve end-users’ experience, reduce costs, improve the experience of clinicians and institutions, and improve the overall health of the population. The Active Compass model presented here is a first step in overcoming some of the obstacles on the long road from bench to bedside. It can assist all those involved in the early stages of product development, and, if implemented correctly, can also be applied in later stages.

## Data Availability

Not applicable.
